# Reliability, criterion-concurrent validity, and construct-discriminant validity of a head-marking version of the taekwondo anaerobic intermittent kick test

**DOI:** 10.5114/biolsport.2022.109459

**Published:** 2021-11-25

**Authors:** Amel Tayech, Mohamed Arbi Mejri, Issam Makhlouf, Aaron Uthoff, Mourad Hambli, David G. Behm, Anis Chaouachi

**Affiliations:** 1 Tunisian Research Laboratory “Sports Performance Optimization”, National Center of Medicine and Science in Sports (CNMSS), Tunis, Tunisia; 2 High Institute of Sport and Physical Education, Ksar-Saïd, Manouba University, Tunis, Tunisia; 3 AUT University, Sports Performance Research Institute New Zealand, Auckland, New Zealand; 4 School of Human Kinetics and Recreation, Memorial University of Newfoundland, St. John’s, Newfoundland, Canada; 5 High Institute of Sport and Physical Education, Sfax University, Sfax, Tunisia

**Keywords:** Martial arts, 30-s continuous jump test, Elite athletes, Smart electronic scoring system, Sport-specific performance, Sensitivity

## Abstract

This study aimed to examine the reliability and validity of a head-marking version of the taekwondo anaerobic intermittent kick test (TAIKT-head). Twenty-seven (21 males and 6 females) taekwondo athletes performed TAIKT-head on two occasions (test-retest). In addition, they performed the chest-marking version of the TAIKT (TAIKT-chest), 30-s continuous jump (CJ30s), countermovement jump (CMJ) and flexibility tests. To establish TAIKT-head’s construct validity (discriminatory capability), two subgroups were identified based on their international and national taekwondo results: 15 elite (12 males and 3 females) and 12 sub-elite (9 males and 3 females) athletes. TAIKT-head showed high relative (ICCs ≥ 0.90) and absolute (SEMs < SWCs) reliability. The comparison between TAIKT-head and TAIKT-chest revealed that absolute and relative peak and mean powers were higher (p < 0.001) in TAIKT-head than in TAIKT-chest. In contrast, the fatigue index and rating of perceived exertion were lower in TAIKT-head than in TAIKT-chest (p < 0.001 and p < 0.01, respectively), with no significant difference between the two tests regarding physiological variables. Significant correlations between TAIKT-head and TAIKT-chest (r ranged from 0.74 to 0.53), CJ30s (r ranged from 0.84 to 0.43), and CMJ (r ranged from 0.88 to 0.79) were mostly “very large”. There was no association between TAIKT-head and flexibility tests. Elite athletes showed greater TAIKT-head performances than sub-elite counterparts. Receiving operating characteristic analysis indicated that the TAIKT-head effectively discriminated between elite and sub-elite athletes. In conclusion, the TAIKT-head is a reliable and valid test to evaluate the specific intermittent anaerobic power of taekwondo athletes through the most used kicking technique at the head level.

## INTRODUCTION

The assessment of physical and physiological qualities through sport specific, scientifically validated, sensitive, and reliable testing [1, 2] can help in identifying the strengths and weaknesses in young and elite athletes to be used for training purposes [3], and thereby provide targeted training to increase the likelihood of success [1]. Recently, Tayech et al. [4, 5] validated a new “Taekwondo Anaerobic Intermittent Kick Test” (TAIKT). The purpose of this test was the assessment of the specific intermittent anaerobic power (individual 5-s sets) and capacity (6 sets of 5-s kicks with 10-s recovery periods) of taekwondo athletes while using new smart technology (i.e., Electronic Body Protector Scoring System (PSS)) [6–9]. The TAIKT focuses on the specific performance properties (i.e., short bursts of high-intensity activity interspersed with low-intensity movements) of taekwondo as an Olympic combat sport [4, 5, 10–12]. The main technical action executed during taekwondo competition is the “Bandal-Tchagui”, or roundhouse kick (≈56.5%) to score in taekwondo competitions, due to its speed and accuracy in kicking the opponent’s target zone [6, 9]. Therefore, it is no surprise that this striking technique has been utilized in many taekwondo-specific high-intensity tests [13–16].

In 2009, the introduction of the PSS for judging taekwondo scores during official competition changed fighting and training methods [6, 17, 18]. An additional smart electronic device (electronic headgear) was introduced for the first time in the 2016 Rio Olympics [6]. The introduction of electronic scoring equipment (electronic body protector and headgear) with integrated wireless sensors and adjusted according to the sex, category and body mass of the athletes by the world Taekwondo was intended to increase the accuracy and fairness of the scoring process by reducing the potential for controversial decisions by referees while providing a more objective assessment during competitions [6–9]. The score points differ depending on the areas of attack and technique use [9]. While only ≈19.8% of attacks are to the head [6], the latest changes in taekwondo competition rules reward valid kicks to the head to score more points compared to kicks to the chest [7–9]. Accordingly, these new rules have forced the athletes to be more active, powerful and accurate when projecting kicks to the head level [6].

Given the above considerations, the use of new smart technologies and rule changes have remarkably influenced the nature of taekwondo competitions [6]. Consequently, the coaches and players have adopted new match tactics and strategies (e.g., multiple kicks to the head) in response to the new scoring system [6, 7, 9].

Based on this notable evolution of taekwondo (i.e., smart electronic headgear, new rules, match strategy), the adjustment of TAIKT to the new technical skill requirements (i.e., the relevance of the kicks to the head) and playing rules (i.e., the introduction of the electronic headgear scoring system) comes about as a necessity to help coaches and sports scientists to rationally and objectively assess the high-intensity intermittent physical performance (i.e., specific intermittent anaerobic power) of taekwondo athletes, especially before the postponed 2021 Olympics. Thus, this study proposes a head-marking version of TAIKT (TAIKT-head) with the aims to: i) examine its reliability; ii) explore its criterion-concurrent validity; iii) establish its relationship with the chest-marking version of TAIKT (TAIKT-chest) [4, 5, 10–12], jumping, and flexibility capabilities; iv) determine whether this test would be sensitive and allow to discriminate between taekwondo athletes of different competitive levels (i.e., elite and sub-elite). We hypothesized that TAIKT-head would show a high level of reliability, validity, and sensitivity, as well as a significant association with indicators of athletic performance.

## MATERIALS AND METHODS

### Subjects

Based on data from Tayech et al. [5], an a priori power analysis using G*Power version 3.1 indicated that a total sample of 19 subjects would be required to detect a large correlation (*r* = 0.60) with 80% power and an alpha of 5%. Twenty-seven elite taekwondo athletes including 21 males and 6 females belonging to the Tunisian taekwondo national team voluntarily participated in this study (Table 1), which took place during the competitive phase. They were regularly competing at a national level for > 7 years and international level for > 5 years. They trained nine sessions of about two hours each per week. Based on their international and national taekwondo performance, 15 athletes were included in the elite and 12 athletes in the sub-elite subgroups, respectively (Table 1). After receiving a thorough explanation of the protocol, as well as the benefits and risks of the investigation, athletes/legal representatives gave written consent to participate in this study. The study was conducted in accordance with the declaration of Helsinki and the protocol was fully approved by the ethics committee of the national center of medicine and sciences in sport of Tunisia before the commencement of the assessments.

**TABLE 1:** Characteristics of subjects participating in the different parts of the study.

Parts of the study	Participants' distribution (n)	Age (year)	Height (cm)	BM (kg)	BMI (kg · m^-2^)	BF (%)	Experience (year)
Reliability and criterion-concurrent validity of TAIKT-head, CJ30s, HAF, and S&R	21 M	19.0 ± 2.0	181.9 ± 5.7	64.3 ± 8.6	19.4 ± 2.3	5.9 ± 2.4	10.4 ± 3.6
	6 F	17.2 ± 0.4	165.3 ± 2.3	54.3 ± 2.3	19.9 ± 1.4	22.2 ± 3.9	7.3 ± 2.0

Construct-discriminant validity of TAIKT-head	15 Elite [12M, 3F]	19.0 ± 2.0	180.6 ± 8.8	64.8 ± 9.9	19.8 ± 2.3	9.3 ± 7.2	10.6 ± 3.4
	12 Sub-elite [9M, 3F]	18.0 ± 1.7	175.2 ± 7.8	58.8 ± 6.1	19.2 ± 1.8	9.8 ± 8.0	8.7 ± 3.5

Total	27	18.6 ± 1.9	178.2 ± 8.7	62.1 ± 8.8	19.5 ± 2.1	9.5 ± 7.4	9.7 ± 3.5

Weight categories	M	-54kg (n = 4), -58kg (n = 5), -63kg (n = 6), -68kg (n = 1), -74kg (n = 4), -80kg (n = 1)					
	F	-49kg (n = 1), -53kg (n = 2), -57kg (n = 2), -62kg (n = 1)					

Values are presented as mean ± standard deviation (SD); TAIKT: Taekwondo Anaerobic Intermittent Kick Test; CJ30s: 30-s continuous jump test; HAF: hip adductor flexibility test; S&R: stand-and-reach test; M: male; F: female; BM: body mass; BMI: body mass index; BF: body fat.

### Procedures

During the week preceding the experiment, participants were familiarized twice with the experimental testing procedures to negate learning effects. They were asked to refrain from strenuous physical activity 48 h prior to the testing days. All testing sessions were conducted indoor at the same time-of-day (between 4 pm and 6 pm), and in the same environmental conditions (26°C and 59% humidity), in randomized and counterbalanced order. The TAIKT-head was performed by elite and sub-elite taekwondo athletes to establish its discriminative ability (construct-discriminant validity). The reliability of the undertaken tests (TAIKT-head, 30-s continuous jump [CJ30s], hip adductor flexibility [HAF], and stand-and-reach [S&R]) was established by means of test-retest trials separated by one week. Prior to all tests, a 15-minute standardized general warm-up (jogging, squatting, jumping, and static and ballistic stretching) was conducted [4, 5]. Prior to the TAIKT-head and TAIKT-chest, the standardized general warm-up was completed with a specific warm-up. Heart rate (HR) was measured every 5-s during each test (TAIKT-head, TAIKT-chest, and CJ30s) using Polar HR monitors (Polar Team^2^ Pro, Kempele, Finland). The blood lactate concentration [La⁻] was recorded 3-min post-test using the Lactate Pro Analyzer (Arkray, Tokyo, Japan), which was calibrated before each measurement according to the manufacturer’s manual. The rating of perceived exertion scale (RPE) [20] of each testing session was recorded immediately after the end of each test (TAIKT-head, TAIKT-chest, and CJ30s) [21]. Standard verbal encouragement was consistently given for all participants throughout the tests by the same researchers.

### Measurements

#### Head-marking version of the taekwondo anaerobic intermittent kick test

**FIG. 1:**
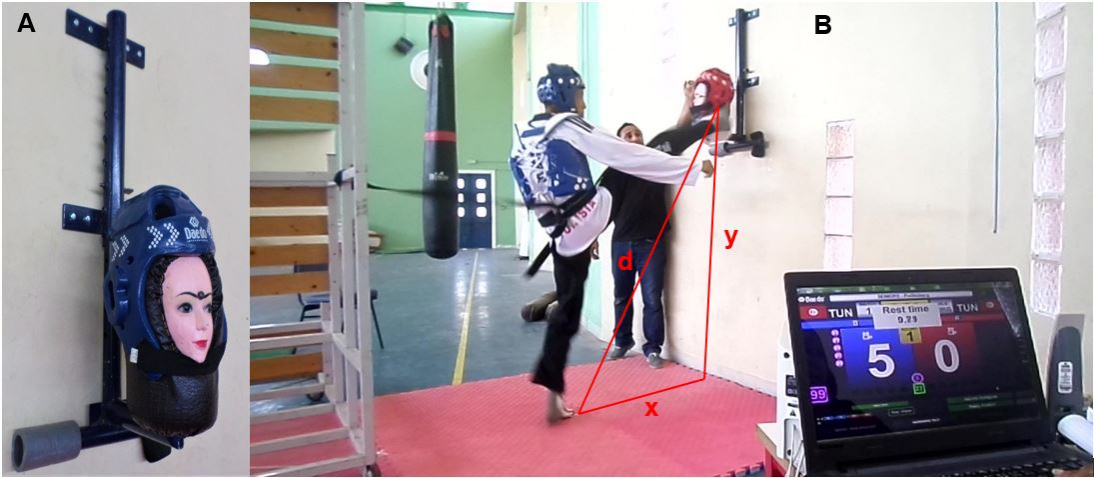
A: Gen-2 E-Headgear worn by the dummy; B: TAIKT-head Protocol. Gen-2 E-Headgear: generation-2 of the electronic headgear; TAIKT-head: Head-marking version of the Taekwondo Anaerobic Intermittent Kick Test; x: distance between the foot and the vertical projection of the contact point of the athlete’s foot on the E-Headgear; y: distance of the vertical projection of the contact point of the athlete’s foot; d: distance between the foot and the E-Headgear (d = √x^2^ + y^2^).

The TAIKT-head protocol is similar to the TAIKT-chest [4, 5, 10, 11], except that in the TAIKT-head the six sets of 5-s successive round-house kicks are projected on a dummy’s head (“Eulgoul” level), alternating right and left legs. The six kicking sets were interspersed with 10-s active recovery (i.e., very light [tempo = one bounce/s] bouncing movements controlled by an assessor). As has been reported by Tayech et al. [4], the 5-s/10-s temporal structure of TAIKT-head was chosen according to the average time of attack or counter-attack during the taekwondo match (i.e., ≈5 seconds) and according to the regular time allocated to the taekwondo athlete during the situation of waiting for the taekwondo combat to continue (i.e., 10 seconds). As in TAIKT-chest and CJ30s [22], the total time for kicks execution during the TAIKT-head was 30-s. The kicks were executed on an electronic headgear (Gen-2 E-Headgear, TK-Strike-Protector, Daedo, Barcelone, Spain) worn by the dummy (Figure 1A), at the same level of the participant’s head, at a height (y) relative to the mat (Figure 1B). This generation-2 of the smart PSS (PSS-G2) (i.e., E-Headgear) only detects kicks to valid scoring areas of the headgear. The sensors in the E-Headgear and on the upper side of the foot protector worn by the participants automatically transmit the score (i.e., the number of validated kicks) to the computer screen when they receive sufficiently strong pressure together [6–9].

Before starting the test, participant adopts the ready stance (“Naranhi Seogi” posture). During kick execution, the participants should not exceed a mark with adhesive tape on the mat, and they were restrained by a taekwondo belt at their hip to prevent them from passing this mark (Figure 1B). This mark is the optimum distance (x) chosen by each participant, to effectively execute kicking sets on the E-Headgear (the bottom of the ears) (Figure 1B) [19]. The distances (x) and (y) (Figure 1B) allow the assessor to determine the distance (d) using the Pythagorean Theorem (d = √x^2^+y^2^) [4, 5, 10], which is the projection distance of the foot on the E-Headgear. This distance (d) was used to calculate the speed and acceleration of each kicks set and allows the assessor to establish the power of each kicks set, according to the lower limb mass (LLM) [4]. As has been previously reported [4, 10], the LLM (kg) was calculated based on the Plagenhoef et al. [23] method. TAIKT-head performances were expressed as absolute (W) and relative (W · kg^-0.67^) peak (P_peak-TAIKT-head_) and mean (P_mean-TAIKT-head_) powers, and fatigue index (FI_TAIKT-head_) (%) [4, 5, 10].

– P_peak-TAIKT-head_ (W): highest power output of the six sets of kicks (anaerobic power);
– P_mean-TAIKT-head_ (W): sum of powers of six sets of kicks/6 (anaerobic capacity);
– FI_TAIKT-head_ (%): [(P_peak-TAIKT-head_-minimum power [P_min-TAIKT-head_])/P_min-TAIKT-head_] × 100.

The absolute power (P) of each kicks’ set was determined using the following formula:

– P (W) = LLM × (d × Nkicks)^2^/(5-s)^3^ [4, 10];
– LLM (kg) = ((thigh+lower leg+foot percentages) × body mass (kg))/100 [4, 10, 23];
– d × Nkicks is the total distance (m) of each kicks’ set;
– 5-s is the execution duration (s) of each kicks’ set;

The relative power of each kicks’ set was calculated with the following formula:

$$\text{P}(\text{W}\cdot {\text{kg}}^{-0.67})=\text{P}(\text{W})/{\text{kg}}^{0.67}$$

[4, 5, 10, 24].

Before performing the test, and following the standardized general warm-up, the participant performed a specific warm-up, including basic kicks (two sets of 20 alternative “Bandal-Tchagui-Montong” and three sets of 20 alternatives “Bandal-Tchagui-Eulgoul”) projected on a taekwondo kick pad, at a moderate rhythm. Five minutes of passive recovery was allowed before performing TAIKT-head.

#### 30-s continuous jump test

The CJ30s is a reliable specific practical test for kicking combat sports, which provides the assessment of the anaerobic power, and explosive power expressed with stretch-shortening cycle movements [22]. This test consisted of maximal continuous vertical jumps performed for 30-s. Participants were required to keep the trunk as vertical as possible, and hands were held akimbo. Participants were asked to flex their knees at ≈90° in the transition between negative/positive phases. The CJ30s was preceded by a specific warm-up composed of five joint mobility exercises (one set of 10-s) with emphasis on the lower limbs and two jumps with 1-min recovery [22]. Verbal feedback was consistently provided to the participants during the test to encourage them to maintain maximum performance until the end of the test. All jumps were assessed with an infrared jump system (Optojump Next instrument, Version 1.3.20.0, Microgate, Bolzano, Italy) interfaced with a microcomputer. Three indices of anaerobic performance expressed as mean power of the first four jumps (P_MEAN_4J_), mean power of all jumps (P_MEAN_) and fatigue index (FI) were calculated. P_MEAN_4J_ and P_MEAN_ were expressed as absolute values (W) and relative to the 0.67^th^ power of body mass (W · kg^-0.67^) [5, 24]. The FI was obtained by considering the first (P_MEAN_4J_) and the last (P_MEAN_end4J_) four jumps of the test, according to this equation: FI(%) = [(P_MEAN_4J_-P_MEAN_end4J_)/P_MEAN_4J_] × 100.

#### Countermovement jump test

As previously described [25], the CMJ test consisted of a vertical jump with the hands akimbo. Three trials were performed, with ≈60-s of rest between jumps, and the best performance was recorded for further analysis.

#### Hip adductor flexibility test

To measure hip adductors’ flexibility, the participant laid in a supine position on the taekwondo mat with hips flexed at 90° and legs elevated against a wall, positioned together with knees extended. The arms are spread laterally at shoulder level [26]. The spreading movement of the legs was performed actively by the participant, who pushed their thighs toward the ground with their hands positioned immediately above the knees so that the hips were maintained in maximum external rotation, and maintained that position for three seconds. The three anatomical points to measure the joint angle of hip were pubic bone and medial malleolus of the fibular bone of right and left leg [26]. For performance measurement (joint angle of hip), all participants’ trials were photographed with a digital camera (high-speed Casio Exilim Ex-zr100 Digital Camera) placed three meters away, perpendicular to hip joint and at a height of one meter. The test was performed three times with 1-min rest between the trials. The photos were downloaded to a computer and subsequently analyzed using openlicense video analysis software (Kinovea 0.8.15 for Windows; available at http://www.kinovea.org). In the current study, the reliability intraclass correlation coefficient (ICC) and standard error of measurement (SEM) of this test were 0.97 and 0.59^°^, respectively.

#### Stand-and-reach test

The S&R was performed as a reliable measure of spinal and pelvic flexibility from the standing position with feet together on a digital forward flexmeter (T.K.K.5403; Takei Scientific Instruments Co., Ltd.) [27]. While keeping the knees, arms and fingers extended, participants were asked to flex at the hips using their maximal range of motion. Three measuring trials with 1-min rest in-betweens were conducted. The average of the best two trials was included into further analyses [27]. The ICC and SEM of S&R were 0.99 and 0.19 cm, respectively.

### Statistics

Two statistical software packages, SPSS 20 (for Windows, Inc., Chicago, IL, USA) and MedCalc (Version 14.8-1993-2014 MedCalc Software) were used for data analyses. Data are presented as means and standard deviations (SD).

To compare the values between the test and retest performance, the paired *t*-test was used when parametric assumption was confirmed by the Kolmogorov-Smirnov test. An unpaired *t*-test was used to compare performances of elite and sub-elite subgroups.

To determine the relative reliability between the test and retest, the ICC was used. An ICC < 0.40 was considered as “low”, between 0.40 and 0.70 as “acceptable”, between 0.70 and 0.90 as “good”, and > 0.90 as “excellent” [28].

Absolute reliability was analysed by calculating the SEM as follows: SEM = SD × √1-ICC [2]. The smallest worthwhile change (SWC) was assumed by multiplying the between-subject SD by either 0.2 (SWC_0.2_), indicating the typical small effect or 0.6 (SWC_0.6_), showing an alternative medium effect or 1.2 (SWC_1.2_), representing an alternative large effect [25]. The usefulness of each test was assessed by comparing the SWC score with the SEM [2]. The ability of the test to detect a change was rated as “good,” “satisfactory,” or “marginal” when the SEM was below, similar, or higher than the SWC, respectively. The minimal detectable change (MDC95%) of TAIKT-head outcomes which represent 95% confidence interval (CI) of the difference in the score between paired observations was determined as MDC95% = 1.96 × SEM × √2. This indicator is interpreted as the minimal change required for a given variable for the assessor to be confident that a real change occurred [1, 2, 25]. Effect size (*d*_z_) for significant pairwise comparisons was calculated using Cohen’s *d* [29] and interpreted as *d*_z_(0.01) = “very small”, *d*_z_(0.2) = “small”, *d*_z_(0.5) = “medium”, *d*_z_(0.8) = “large”, *d*_z_(1.2) = “very large”, and *d*_z_(2.0) = “huge” [25].

The criterion-concurrent validity of the TAIKT-head was established by assessing the relationship between TAIKT-head, TAIKT-chest, CJ30s, CMJ, HAF, and S&R outcomes using Pearson’s product moment correlation coefficient (*r*). The following criteria were adopted to interpret the magnitude of the correlation: “trivial” (*r* < 0.1), “small” (0.1 ≤ *r* < 0.3), “moderate” (0.3 ≤ *r* < 0.5), “large” (0.5 ≤ *r* < 0.7), “very large” (0.7 ≤ *r* < 0.9), “nearly perfect” (0.9 ≤ *r* < 1), and “perfect” (*r* = 1) [28]. In line with this scale, criterion-concurrent validity was accepted when a ‘‘large’’ value (or above) was observed between the TAIKT-head and TAIKT-chest, and CJ30s. Coefficient of determination (R^2^) was used to interpret the meaningfulness of the relationships between TAIKT-head and other outcomes [25]. To investigate whether prediction equations may be developed to determine the TAIKT-head performance from TAIKT-chest performance and vice versa, linear regression was used to model the relationship between the variables of these aforementioned two tests [30].

The construct-discriminant validity of the TAIKT-head was analyzed using the receiver operator characteristics (ROC) curve with analyses of the area under the curve (AUC) [5, 25]. The ROC curve analysis determined the sensitivity and specificity of a tool to evaluate the ability of the different tests that can discriminate between athletes of different competitive levels (i.e., elite vs sub-elite) [25]. The cut-off value for a “good” discriminative ability was 0.70. The significance level was set at *p* < 0.05.

## RESULTS

The relative and absolute reliability analyses of the TAIKT-head are displayed in Table 2.

**TABLE 2:** Descriptive performances of subjects, Student’s *t*-test and mean differences for the test–retest complemented with reliability statistics of TAIKT-head outcomes.

		Test (mean ± SD)	Retest (mean ± SD)	Mean difference ± SD	p value	ICC (95% CI) magnitude	SEM	SWC (0.2, 0.6, and 1.2)	MDC95%
P_peak_	(W)	16.30 ± 4.65	16.15 ± 4.53	0.152 ± 1.379	0.572	0.98 (0.951 to 0.990) excellent	0.21	0.92, 2.75, 5.51	0.58
	(W · kg^-0.67^)	1.02 ± 0.22	1.01 ± 0.20	0.011 ± 0.089	0.536	0.95 (0.900 to 0.979) excellent	0.02	0.04, 0.13, 0.25	0.05

P_mean_	(W)	14.45 ± 4.33	14.51 ± 4.21	-0.057 ± 0.817	0.720	0.99 (0.980 to 0.996) excellent	0.08	0.85, 2.56, 5.12	0.21
	(W · kg^-0.67^)	0.91 ± 0.21	0.91 ± 0.19	-0.004 ± 0.053	0.720	0.98 (0.962 to 0.992) excellent	0.01	0.04, 0.12, 0.24	0.02

FI	(%)	25.19 ± 4.25	25.26 ± 4.12	-0.067 ± 0.908	0.704	0.99 (0.975 to 0.995) excellent	0.10	0.84, 2.51, 5.02	0.28

HR_peak_	(bpm)	182 ± 10	182 ± 7	-0.074 ± 4.898	0.938	0.91 (0.800 to 0.959) excellent	1.48	1.64, 4.92, 9.83	4.10

[La⁻]	(mmol · l^-1^)	9.8 ± 2.8	9.6 ± 2.5	0.237 ± 1.171	0.302	0.95 (0.890 to 0.977) excellent	0.26	0.53, 1.60, 3.20	0.73

RPE	–	5 ± 2	5 ± 1	0.111 ± 0.892	0.523	0.87 (0.723 to 0.942) good	0.32	0.26, 0.78, 1.56	0.88

TAIKT: Taekwondo Anaerobic Intermittent Kick Test; P_peak_: peak power; P_mean_: mean power; FI: fatigue index; HR_peak_: peak heart rate; [La⁻]: lactate concentration; RPE: ratings of perceived exertion; p: significations; ICC: intraclass-correlation coefficient; CI: confidence interval; SEM: standard error of measurement; SWC: smallest worthwhile change; MDC95%: minimal detectable change at 95% confidence interval.

All TAIKT-head outcomes were not significantly different between the test and retest (*p* > 0.05) (Table 2). The results suggest that an “excellent” relative reliability (ICC > 0.90) was observed for the TAIKT-head outcomes, except for the RPE which presented a “good” relative reliability.

For absolute reliability, the SEMs were less than SWCs_(0.2,0.6,1.2)_ for all of the variables of TAIKT-head (Table 2), and considered as “good”, except for RPE, with the SEM was less than SWCs_(0.6,1.2)_. The MDCs95% were “acceptable” for all TAIKT-head variables (Table 2).

**FIG. 2:**
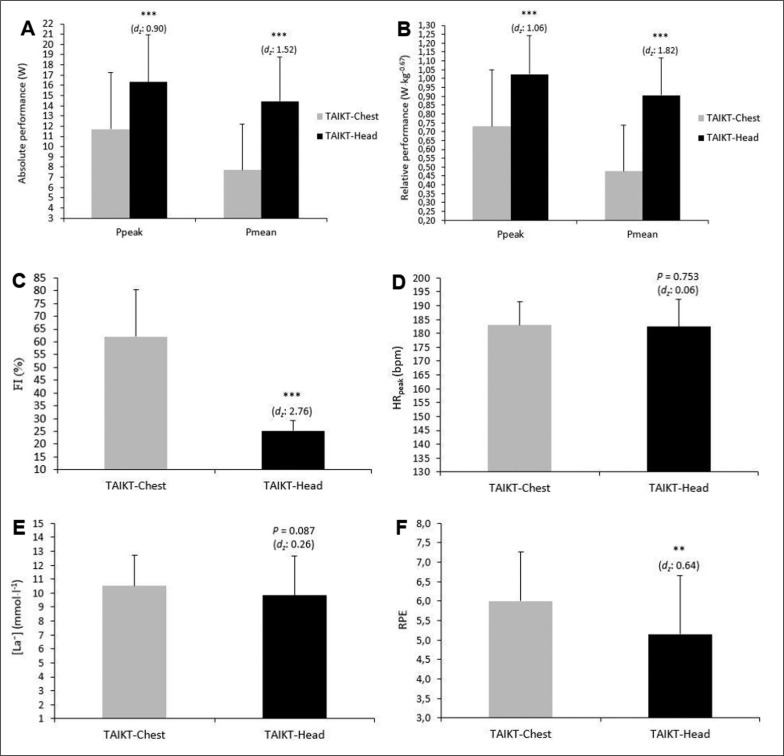
Comparison of performance, physiological and perceptual variables between TAIKT-head and TAIKT-chest. A: comparison of absolute powers (W); B: comparison of relative powers (W·kg^-0.67^); C: comparison of FI (%); D: comparison of HR_peak_ (bpm); E: comparison of [La⁻] (mmol·l^-1^); F: comparison of RPE; d_z_: Cohen’s *d* effect size; ^**^p < 0.01; ^***^p < 0.001.

Comparisons between the TAIKT-head and TAIKT-chest data (Figure 2) showed significant inter-test differences (*p* < 0.001) in P_peak_, P_mean_ (W [A]; W · kg^-0.67^ [B]). However, the FI (C) and RPE (F) values from the TAIKT-chest were significantly larger than corresponding values for the TAIKT-head (*p* < 0.001 and *p* < 0.01, respectively). There were no significant difference for HR_peak_ (*p* = 0.753; *d*_z_ = 0.06) (D) and [La⁻] (*p* = 0.087; *d*_z_ = 0.26) (E) between the tests.

The correlation coefficient, confidence interval, magnitude, and coefficient of determination between athletes’ performances recorded during the TAIKT-head and TAIKT-chest, CJ30s, HAF, and S&R are summarized in (Table 3). Significant correlations (“large” to “very large”) were observed between the TAIKT-head and TAIKT-chest, CJ30s, and CMJ variables. The highest associations obtained were between the P_mean-TAIKT-head_ (W and W · kg-0.67) and P_mean-TAIKT-chest_ (W and W · kg^-0.67^), and P_MEAN-CJ30s_ (W and W · kg^-0.67^), as well as between P_peak-TAIKT-head_ (W and W · kg^-0.67^), and P_MEAN_4J-CJ30s_ (W and W · kg^-0.67^), and P_peak-CMJ_ (W and W · kg^-0.67^). Similarly, the HR-_peak-TAIKT-head_, and [La⁻]_TAIKT-head_ were significantly correlated (“very large magnitude”) with the corresponding HR_peak-CJ30s_, and [La⁻]_TAIKT-chest_, respectively. The “large” associations were found between: i) P_peak-TAIKT-head_ and P_peak-TAIKT-chest_ (W and W · kg^-0.67^), ii) FI_TAIKT-head_ and FI_TAIKT-chest_, and FI_CJ30s_, iii) HR_peak-TAIKT-head_ and HR_peak-TAIKT-chest_, iv) [La⁻]_TAIKT-head_ and [La⁻]_CJ30s_, v) RPE_TAIKT-head_ and RPE_TAIKT-chest_. A “moderate” association was observed between the RPE_TAIKT-head_ and RPE_CJ30s_. There was no significant associations between the P_peak-TAIKT-head_ and HAF, and S&R.

**TABLE 3:** Pearson’s correlation (*r*) of performances, physiological, and perceptual variables between TAIKT-head and TAIKT-chest, CJ30s, CMJ, HAF, and S&R (n = 27).

**TAIKT-head**								

	P_peak-TAIKT-head_		P_mean-TAIKT-head_		FI_TAIKT-head_	HR_peak-TAIKT-head_	[La⁻]_TAIKT-head_	RPE_TAIKT-head_

	(W)	(W × kg^-0.67^)	(W)	(W × kg^-0.67^)	(%)	(bpm)	(mmol × l^-1^)	

Mean ± SD	16.30 ± 4.65	1.02 ± 0.22	14.45 ± 4.33	0.91 ± 0.21	25.19 ± 4.25	182 ± 10	9.8 ± 2.8	5 ± 2

**TAIKT-chest**								

	P_peak-TAIKT-chest_		P_mean-TAIKT-chest_		FI_TAIKT-chest_	HR_peak-TAIKT-chest_	[La⁻]_TAIKT-c hest_	RPE_TAIKT-chest_

	(W)	(W × kg^-0.67^)	(W)	(W × kg^-0.67^)	(%)	(bpm)	(mmol × l^-1^)	

Mean ± SD	11.70 ± 5.59	0.73 ± 0.32	7.69 ± 4.54	0.48 ± 0.26	62.08 ± 18.41	183 ± 9	10.5 ± 2.2	6 ± 1
*r*	0.69	0.74	0.70	0.60	0.72	0.64
*p* value	< 0.001	0.001	< 0.001	< 0.001	0.0049	< 0.001	< 0.001	< 0.001
95% CI	0.42 to 0.85	0.27 to 0.79	0.50 to 0.87	0.43 to 0.85	0.18 to 0.75	0.29 to 0.80	0.47 to 0.86	0.34 to 0.82
Magnitude	large	large	very large	very large	large	large	very large	large
R^2^ (%)	47.85	35.14	54.52	48.75	27.56	36.42	52.05	41.04

**CJ30s**								

	P_MEAN 4J-CJ30s_		P_MEAN-CJ30s_		FI_CJ30s_	HR_peak-CJ30s_	[La⁻]_CJ30s_	RPE_CJ30s_

	(W)	(W × kg^-0.67^)	(W)	(W × kg^-0.67^)	(%)	(bpm)	(mmol × l^-1^)	

Mean ± SD	2763.41 ± 618.24	172.63 ± 27.49	2531.85 ± 582.90	158.07 ± 25.96	17.50 ± 4.57	182 ± 8	6.2 ± 1.7	5 ± 1
*r*	0.84	0.73	0.64	0.74	0.54	0.43
*p* value	< 0.001	< 0.001	< 0.001	< 0.001	< 0.001	< 0.001	0.0039	0.0264
95% CI	0.67 to 0.92	0.48 to 0.87	0.68 to 0.93	0.51 to 0.88	0.34 to 0.82	0.50 to 0.87	0.20 to 0.76	0.06 to 0.69
Magnitude	very large	very large	very large	very large	large	very large	large	moderate
R^2^ (%)	69.91	52.64	70.76	55.75	41.07	54.43	28.74	18.23

**CMJ**								

	P_peak-CMJ_							

	(W)	(W × kg^-0.67^)						

Mean ± SD	187.43 ± 25.41	–	–	–	–	–	–
*r*	0.88	0.79	–	–	–	–	–	–
*p* value	< 0.001	< 0.001	–	–	–	–	–	–
95% CI	0.74 to 0.94	0.58 to 0.90	–	–	–	–	–	–
Magnitude	very large	very large	–	–	–	–	–	–
R^2^ (%)	76.88	62.18	–	–	–	–	–	–

**HAF (°)**								

Mean ± SD	140.3 ± 9.9	–	–	–	–	–	–	–
*r*	-0.15	–	–	–	–	–	–	–
*p* value	0.45	–	–	–	–	–	–	–
95% CI	-0.50 to 0.24	–	–	–	–	–	–	–
Magnitude	small	–	–	–	–	–	–	–
R^2^ (%)	2.31	–	–	–	–	–	–	–

**S&R (cm)**								

Mean ± SD	12.5 ± 6.6	–	–	–	–	–	–	–
*r*	0.12	–	–	–	–	–	–	–
*p* value	–	–	–	–	–	–	–
95% CI	-0.27 to 0.48	–	–	–	–	–	–	–
Magnitude	small	–	–	–	–	–	–	–
R^2^ (%)	1.38	–	–	–	–	–	–	–

TAIKT: Taekwondo Anaerobic Intermittent Kick Test; CJ30s: 30-s continuous jump test; HAF: hip adductor flexibility test; S&R: stand-and-reach test; P_MEAN_4J_: mean power of the first four jumps; P_MEAN_: mean power of all jumps; HR_peak_: peak heart rate; [La⁻]: lactate concentration; RPE: ratings of perceived exertion; P_peak_: peak power; P_mean_: mean power; FI: fatigue index; *r*: Pearson correlation coefficient; 95% CI: 95% confidence interval; R^2^: coefficient of determination.

Analysis of common variance using the R^2^ (Table 3) revealed that the absolute and relative P_peak-CMJ_ and P_MEAN_4J-CJ30s_ accounted for the greatest R^2^ for absolute and relative P_peak-TAIKT-head_, respectively. Moreover, the absolute and relative P_MEAN-CJ30s_ and P_mean-TAIKT-chest_ accounted for the greatest R^2^ for absolute and relative P_mean-TAIKT-head_, respectively. The corresponding regression equations to estimate TAIKT-head from TAIKT-chest performances and vice versa are displayed in Table 4.

**TABLE 4:** Regression equations ± standard error to estimate TAIKT-head from TAIKT-chest indices and vice versa.

TAIKT-head		TAIKT-chest	p value
P_peak_	(W)	9.57 + 0.58×± 1.55	< 0.001
	(W · kg^-0.67^)	0.73 + 0.40×± 0.09	< 0.001
P_mean_	(W)	9.03 + 0.70×± 1.14	< 0.001
	(W · kg^-0.67^)	0.64 + 0.56×± 0.06	< 0.001
FI	(%)	17.67 + 0.12×± 2.54	< 0.01

**TAIKT-chest**		**TAIKT-head**	**P value**

P_peak_	(W)	-1.86 + 0.83×± 2.94	< 0.001
	(W · kg^-0.67^)	-0.16 + 0.87×± 0.25	< 0.01
P_mean_	(W)	-3.49 + 0.77×± 2.13	< 0.001
	(W · kg^-0.67^)	-0.31 + 0.87×± 0.17	< 0.001
FI	(%)	4.75 + 2.28×± 18.84	< 0.01

TAIKT: Taekwondo Anaerobic Intermittent Kick Test; P_peak_: peak power; P_mean_: mean power; FI: fatigue index.

The comparison of TAIKT-head outcomes between elite and sub-elite taekwondo athletes are displayed in Table 5. The unpaired sample *t*-test revealed significantly higher TAIKT-head performances in elite compared to sub-elite athletes. However, no significant between groups differences were observed for HR_peak_, [La⁻], and RPE.

**TABLE 5:** Comparison of performances, physiological and perceptual variables between elite and sub-elite taekwondo athletes during and after the TAIKT-head

		Elite (n = 15) (mean ± SD)	Sub-elite (n = 12) (mean ± SD)	Mean difference ± SD	p value	d_z_
P_peak_	(W)	18.45 ± 4.73	13.62 ± 2.92	4.838 ± 1.482	0.003	-1.20
	(W · kg^-0.67^)	1.13 ± 0.21	0.89 ± 0.16	0.235 ± 0.071	0.003	-1.27
P_mean_	(W)	16.20 ± 4.46	12.26 ± 3.08	3.950 ± 1.455	0.012	-1.01
	(W · kg^-0.67^)	0.99 ± 0.21	0.80 ± 0.17	0.186 ± 0.072	0.016	-0.98
FI	(%)	24.88 ± 5.43	25.58 ± 2.20	-0.699 ± 1.538	0.044	0.16
HR_peak_	(bpm)	181 ± 12	184 ± 7	-2.333 ± 3.678	0.532	0.24
[La⁻]	(mmol · l^-1^)	9.7 ± 2.9	10.0 ± 2.8	-0.295 ± 1.109	0.793	0.10
RPE	–	5 ± 2	5 ± 1	-0.183 ± 0.572	0.515	0.12

TAIKT: Taekwondo Anaerobic Intermittent Kick Test; P_peak_: peak power; P_mean_: mean power; FI: fatigue index; HR_peak_: peak heart rate; [La⁻]: lactate concentration; RPE: ratings of perceived exertion; p: significations; *d*_z_: Cohen’s d.

**FIG. 3:**
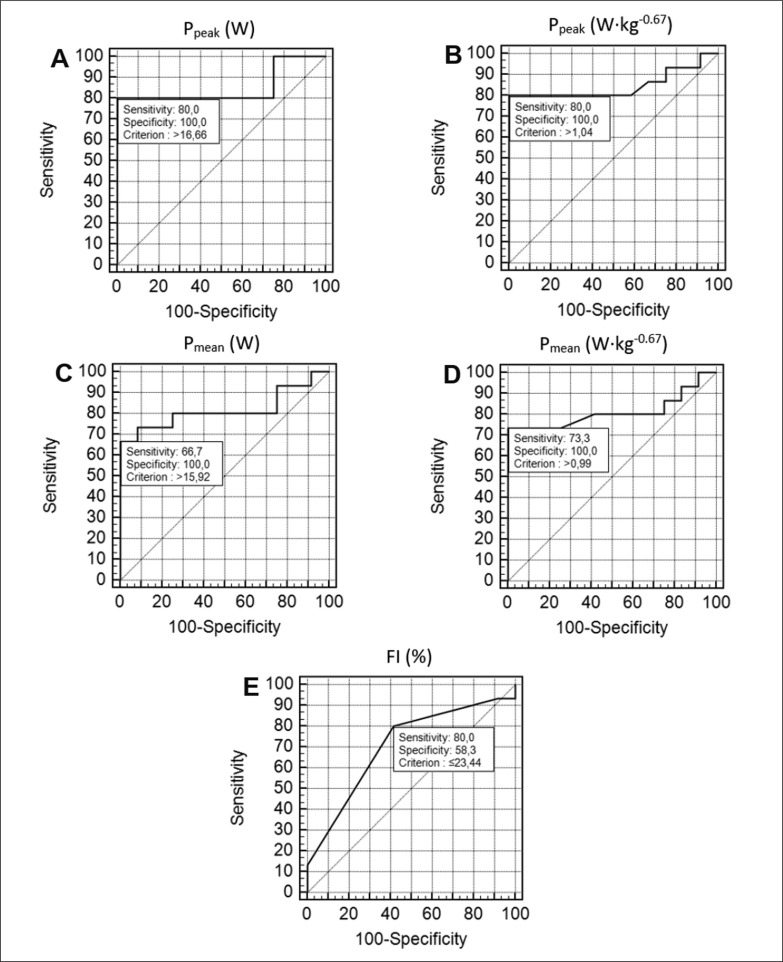
Receiver operating characteristics (ROC) curves for the TAIKT-head performance of elite and sub-elite taekwondo athletes. A: P_peak_ (W); B: P_peak_ (W∙kg^-0.67^); C: P_mean_ (W); D: P_mean_ (W∙kg^-0.67^), E: FI (%).

The TAIKT-head was considered to have very good discriminant validity (Figure 3). The area under the ROC curve (AUC) was 0.85 (SE = 0.084; 95% CI:0.66–0.96, *p* < 0.001) for P_peak_ (W) (A), 0.85 (SE = 0.085; 95% CI:0.66–0.96, *p* < 0.001) for P_peak_ (W · kg^-0.67^) (B), 0.82 (SE = 0.090; 95% CI:0.62–0.94, *p* < 0.001) for P_mean_ (W) (C), and 0.81 (SE = 0.092; 95% CI:0.62–0.94, *p* < 0.001) for P_mean_ (W · kg^-0.67^) (D). The AUC of FI was 0.71 (SE = 0.093; 95% CI:0.50–0.86, *p* = 0.027) (E). The cut-off performances for discriminating between the elite and sub-elite athletes (Figure 3) were > 16.66 W for P_peak_ (W) (A), > 1.04 W · kg^-0.67^ for P_peak_ (W · kg^-0.67^) (B), > 15.92 W for P_mean_ (W) (C), > 0.99 W · kg^-0.67^ for P_mean_ (W · kg^-0.67^) (D), and ≤ 23.44% for FI (%) (E).

## DISCUSSION

This study aimed to assess the reliability and validity of the TAIKT-head and to examine whether this test could be used to distinguish between elite and sub-elite taekwondo athletes. The main findings of this study demonstrated that the TAIKT-head is a reliable and valid test to evaluate the specific intermittent anaerobic power of taekwondo athletes. Furthermore, this taekwondo-specific test showed a very good ability to effectively discriminate between elite and sub-elite taekwondo athletes.

The results of this study showed high relative and absolute reliability of TAIKT-head. Excellent ICCs were found (> 0.90) for absolute and relative P_peak_ and P_mean_, FI, HR_peak_, and [La⁻], with “good” relative reliability for RPE. The ICCs found in the present study were higher than those observed in similar taekwondo-specific high-intensity tests [31, 32]. Indeed, in the study of Rocha et al. [31], the ICCs relating to the test and retest performances of the taekwondo specific anaerobic test (TSAT) (i.e., absolute and relative P_peak_ and P_mean_, and FI) ranged between 0.83 and 0.87. Moreover, Santos et al. [32] found ICCs ≈0.85 relating to the test and retest performances of the multiple-set frequency of the speed kick test. In view of the above findings, it has been reported that the reliability of a test depends on a number of factors such as the number of participants, number of performed trials, participant’s skill level, and homogeneity of the sample [25]. The current study included 27 elite taekwondo athletes belonging to the taekwondo national team. This number exceeded those of participants (n > 17) from previous studies with good relative reliability [31, 32]. Furthermore, as reported by Makhlouf et al. [25], the excellent reliability found in the current study can also be explained by the participants’ test familiarization and the standardized environmental conditions during test and retest sessions.

Absolute reliability refers to the degree to which experienced subjects maintain their position in a sample with repeated measurements with SEM used for evaluative tests to monitor changes over time [2, 4, 5, 14, 25]. In addition, Hopkins [2] proposed that when SEM is higher than SWC, the evaluation of the variable being used was “marginal”, when SEM was similar to the SWC, it was “medium;” and if SEM was less than the SWC, an evaluation of “good” was given to the test to detect small (0.2), medium (0.6), and large (1.2) differences. In summary, in the current study, the absolute reliability of the TAKT-head variables, expressed as the SWCs_(0.2,0.6,1.2)_ were higher than SEMs, so the evaluation was “good” and allows the test to detect, “small” changes in performances. These results show that TAIKT-head effectively helps taekwondo athletes to improve their specific anaerobic performance, with a more precise measure. In addition to reliability, it is important to understand the amount of change required to be certain that the change in TAIKT-head performances is not due to measurement error. In this regard, when a change in TAIKT-head outcomes exceed the MDCs95% values, a true change could be considered “real” reflecting a true performance improvement in elite-level taekwondo athletes [1, 4, 13].

When comparing TAIKT-head and TAIKT-chest outcomes, we noticed that the absolute and relative P_peak_ and P_mean_ were higher (*p* < 0.001) in TAIKT-head compared to the TAIKT-chest. In contrast, the FI and RPE were lower in TAIKT-head than in TAIKT-chest. from the same-perspective, Hachana et al. [24] found that absolute, relative, and derived P_peak_ and P_mean_ were significantly higher in 15s-WAnT than in 30s-WAnT, and conversely the FI was significantly lower in 15s-WAnT than in 30s-WAnT. This latter result can be explained by the fact that the roundhouse kick to the head level has been reported to have a higher velocity and impact force compared to a roundhouse kick to the chest level [33, 34]. Indeed, it has been reported that successive kicks to the head following each strike, require stretch shortening cycle (SSC) capabilities, where the leg is quickly driven back down into the ground and then quickly driven back up toward the target [34]. It is well documented that efficient SSC mechanics result in enhanced propulsive force [35] translating to increased striking forces due to increased energy return [36] and conservation of energy [37]. Turner [34] reported that the optimisation of SSC mechanics during a sequence of successive kicks to the head, requires that ground contact should be minimised [38], promoting an increased rate of force development [39] and reducing the duration and metabolic cost of movement [35, 37]. This may help explain the high powers (P_peak_ and P_mean_) and the low FI recorded during TAIKT-head, as well as the highest correlations found between TAIKT-head and CMJ performances, in the current study. The present findings greatly exceeded the correlations (*r* ranged from 0.56 to 0.59) between TSAT and CMJ outcomes found in the study by Rocha et al. [31], as well as the correlations (*r* = 0.70) between the specific taekwondo anaerobic test and CMJ outcomes found in the study by Sant’Ana et al. [40]. There was no significant difference between TAIKT-head and TAIKT-chest regarding HR_peak_, and [La⁻] values. These results are in line with those recorded by Tayech et al. [5] (i.e., HR_peak_ ≈182 vs. 188 bpm and [La⁻] ≈10 mmol × l^-1^). Accordingly, the high HR_peak_ and [La⁻] values clearly pointed to the maximal intensity of the TAIKT-head with similar values as those recorded during taekwondo matches [21, 41].

Pearson’s correlation analysis revealed a “large” to “very large” relationship between the TAIKT-head and TAIKT-chest in all performance, physiological, and perceptual variables. This strong correlation provided criterion-concurrent validity to the TAIKT-head. The R^2^ showed that TAIKT-head and TAIKT-chest shared high common variances in taekwondo athletes. Accordingly, regression analyses showed that TAIKT-head performance could be partially predicted from TAIKT-chest performance and vice versa. Indeed, depending on the availability of the specific taekwondo equipment (i.e., E-headgear or PSS), the coaches can perform the appropriate test (i.e., TAIKT-head or TAIKT-chest), and then extrapolate the required performance from the other test.

Criterion-concurrent validity has generally been studied by associating the sport-specific test outcome with a gold standard protocol based on actions involving specific muscle groups of this sport [1]. According to Nikolaidis et al. [42] neuromuscular capacity, including jumping ability is a main determinant of anaerobic fitness in taekwondo athletes. Findings of this study showed “large” to “very large” correlation coefficients between the TAIKT-head and CJ30s in all performance and physiological variables; corroborating the findings of Rocha et al. [31] who demonstrated that the TSAT was highly correlated (magnitude ranged from “large” to “very large”) with the 30s-WAnT in taekwondo athletes. In addition, Oliveira et al. [43] obtained significant correlations (*r* ranged between 0.31 and 0.86) in the comparison between the 30s-WAnT and adapted anaerobic kick test results in taekwondo athletes. A “moderate” relationship was observed between the RPE_TAIKT-head_ and RPE_CJ30s_. The R^2^ showed that the TAIKT-head and CJ30s performance shared high common variances in taekwondo athletes. Evidence of the anaerobic nature of the TAIKT-head has been supported by the magnitude of the correlation coefficient between the TAIKT-head and the CJ30s [La⁻] values (*r* = 0.54, *p* = 0.0039) [44]. Accordingly, the strong correlations between TAIKT-head and CJ30s strengthen the criterion-concurrent validity of the TAIKT-head.

Flexibility plays an important role in taekwondo competition [45] to enable athletes to project high kicks to the opponent’s head [46]. HAF and S&R were the most used flexibility tests in the specialized taekwondo studies, to test the flexibility capability among taekwondo athletes [47–49]. In the current study, a “small” association between the TAIKT-head and flexibility performance (HAF and S&R) was found. Accordingly, taekwondo elite athletes do not have to be extremely flexible; nevertheless, an optimal level of flexibility is required so as not to interfere with their strength and power capabilities. Indeed, it has been demonstrated that high flexibility levels are deleterious to skills requiring high strength or power levels [50].

In accordance with Tayech et al. [5] findings, the study’s results found that elite athletes showed greater TAIKT-head performances compared with sub-elite athletes. However, no significant differences were found between the two groups in physiological and perceptual variables. Using the ROC method [1], the TAIKT-head showed a “very good” ability to effectively discriminate between elite and sub-elite taekwondo athletes. The findings of the current study are similar to those found by Chaabene et al. [13] and Tayech et al. [5], where significant differences between performances of elite and sub-elite taekwondo athletes in favour of the elite were observed through the new taekwondo-specific tests. Based on the above results, coaches could use the TAIKT-head to distinguish between taekwondo athletes of different competitive levels.

A limitation of the present study is that the sample included two levels of competitive athletes only (elite and sub-elite) and a limited number of female athletes (6 females). Therefore, further investigations should check the reliability and validity of the TAIKT-head among taekwondo athletes of different ages, sex and athletic levels [19]. Despite this limit, the present findings provide a valuable opportunity for the assessment of high-intensity intermittent physical performance of taekwondo athletes. Additional research is needed to show whether this specific test is sensitive enough to monitor the small changes in performance among taekwondo athletes during the season.

## CONCLUSIONS

The TAIKT-head offers a reliable and valid tool for measuring the specific intermittent anaerobic power of taekwondo athletes through the most used kicking technique at the head level, while responding to the new requirements and rules in taekwondo competitions. This tool could be used as specific test for Olympic foot striking combat sports, since it is characterized by a good ability to discriminate between elite taekwondo athletes of different competitive levels.
